# Induction of Proinflammatory Cytokines and C-Reactive Protein in Human Macrophage Cell Line U937 Exposed to Air Pollution Particulates

**DOI:** 10.1289/ehp.8094

**Published:** 2005-07-21

**Authors:** Christoph Franz Adam Vogel, Eric Sciullo, Pat Wong, Paul Kuzmicky, Norman Kado, Fumio Matsumura

**Affiliations:** 1 Department of Environmental Toxicology, University of California, Davis, California, USA; 2 California Environmental Protection Agency, Air Resources Board, Sacramento, California, USA; 3 Center for Environmental Health Sciences, University of California, Davis, California, USA

**Keywords:** aryl hydrocarbon receptor, cyclooxygenase-2, C-reactive protein, cytokines, foam cells, interleukin-8, inflammation, macrophages, particles, 2,3,7,8-tetrachlorodibenzo-*p*-dioxin

## Abstract

Exposure to particulate matter air pollution causes inflammatory responses and is associated with the progression of atherosclerosis and increased cardiovascular mortality. Macrophages play a key role in atherogenesis by releasing proinflammatory cytokines and forming foam cells in subendothelial lesions. The present study quantified the inflammatory response in a human macrophage cell line (U937) after exposure to an ambient particulate sample from urban dust (UDP) and a diesel exhaust particulate (DEP). The effect of native UDP and DEP was compared with their corresponding organic extracts (OE-UDP/OE-DEP) and stripped particles (sUDP/sDEP) to clarify their respective roles. Exposure to OE-UDP, OE-DEP, UDP, DEP, and 2,3,7,8-tetrachlorodibenzo-*p*-dioxin led to a greater increase of interleukin (IL)-8, tumor necrosis factor-α, and cyclooxygenase-2 mRNA expression than did the stripped particles, whereas sUDP, sDEP, UDP, and DEP led to a greater production of C-reactive protein and IL-6 mRNA. The particles and the organic extract-induced expression of cyclooxygenase-2 and cytochrome P450 (CYP)1a1 was significantly suppressed by co-treatment with an aryl hydrocarbon receptor (AhR) antagonist, indicating that these effects are mainly mediated by the organic components, which can activate the AhR and CYP1a1. In contrast, the induction of C-reactive protein and IL-6 seems to be a particle-related effect that is AhR independent. The inflammatory response induced by particulate matter was associated with a subsequent increase of cholesterol accumulation, a hallmark of foam cells. Together, these data illustrate the interaction between particulate matter and the inflammatory response as well as the formation of cholesterol-accumulating foam cells, which are early markers of cardiovascular disease.

Numerous epidemiologic studies have observed that exposure to particulate matter (PM) air pollution, which occurs in many urban and industrial environments, is associated with an increase of cardiovascular diseases and mortality (Brook et al. 2003; Morris 2001). Although the exact components of PM and the exact mechanism leading to cardiovascular disease and cardiopulmonary disease mortality from exposure to PM are still unknown, several studies have shown that systemic inflammation may be a key step in these pathological processes through inflammatory mediators (Salvi et al. 1999) such as cyclooxygenase-2 (COX-2), interleukin (IL)-1β, and tumor necrosis factor-α(TNFα), which are among the most important mediators of the inflammatory response (Ross 1999) in the development of atherosclerotic vascular disease (Libby 2002). A positive correlation of C-reactive protein (CRP) and coronary artery disease, which could be explained by the atherogenic effects of chronic inflammation, is well known (Beck et al. 1999; Mendall et al. 1996). Recently, an association between minor but chronic elevation of serum CRP levels and future major cardiovascular events has been shown (Yeh 2004). Elevated levels of proinflammatory cytokines and CRP play a significant role in the genesis of atherosclerosis and in plaque instability (Libby 2002). CRP activates complement through binding to the Fcγ receptor and enhancing phagocytosis of low-density lipoprotein, leading to the formation of foam cells (Zwaka et al. 2001), thus directly contributing to the development of atherosclerosis. Despite the epidemiologic evidence, experimental verification of the causal relationship among air pollution, CRP, and cardiovascular disease is still limited.

*In vitro* studies show increased levels of proinflammatory cytokines including TNFα, IL-1β, IL-6, and IL-8, which have been described in various cell types after exposure to PM (Mathiesen et al. 2004; Monn et al. 2003; Monn and Becker 1999; van Eeden et al. 2001). Acute exposure to diesel exhaust also increased IL-8 production in human airways (Salvi et al. 2000). However, elevated serum levels of CRP, the classic acute-phase protein, has only been found to be associated with exposure to an elevated concentration of PM in humans (Kim et al. 2005; Peters et al. 2001; Pope et al. 2004a; Seaton et al. 1999). Other harmful effects described by these authors involved the triggering of acute vasoconstriction and the development of atherosclerosis. A few animal models have shown the harmful effects of inhalation of air pollutants on cardiovascular functions (Campen et al. 2003; Gordon et al. 1998), as well as on the etiology of atherosclerosis (Suwa et al. 2002). Suwa et al. (2002) showed that exposure of rabbits to PM_10_ (PM with areodynamic diameter ≤10 μm) causes progression of atherosclerotic lesions, and a number of alveolar macrophages phagocytosed PM_10_. Direct effects of PM may occur via components that are able to cross the pulmonary epithelium into the circulation, such as gases, ultrafine particles (Nemmar et al. 2002), and soluble co-pollutants (e.g., polycyclic aromatic hydrocarbons and transition metals).

To clarify the contribution of each component of PM in the induction of the inflammatory response, we systematically compared the effects induced by PM derived from different sources such as diesel exhaust particulates (DEP) and urban dust particulates (UDP) with those induced by their organic extracts OE-DEP/OE-UDP and the fine particles or coarse fraction, represented by their stripped particles sDEP/sUDP and the ultrafine particles carbon black (CB).

The present study provides evidence that the organic components of the native particles DEP and UDP play a major role in mediating the increase of the inflammatory cytokines TNFα, IL-8, and COX-2. We also demonstrate, for the first time, an increased expression of CRP in macrophages induced by the particles that is mediated by the particulate composition rather than their organic components.

## Materials and Methods

### Reagents.

National Institute of Standards and Technology (NIST) Standard Reference Material (SRM) 1649, an atmospheric particulate material collected in an urban area, and a diesel exhaust particulate sample, NIST SRM 2975, were purchased from NIST (Gaithersburg, MD). CB 95 nm in diameter (FR103) were provided by Degussa (Frankfurt, Germany). We prepared stock solutions of particles by suspending them in autoclaved distilled water and by ultrasonication for 2 min at maximum power (100 W). Particles were used at 2.5, 10, or 40 μg/cm^2^, equivalent to 12.5, 50, or 200 μg/mL. Concentrations are preferentially expressed in micrograms per square centimeter because particles rapidly sediment onto the cell layer. UDP and DEP were extracted by dichloromethane in a soxhlet apparatus. After sonication the extract was filtered (0.45 μm Acrodisc) and concentrated to 1 mL by TurboVap and stored in precleaned amber vials. The extract obtained was dried and then redissolved in dimethylsulfoxide. We used the OE-DEP and OE-UDP at concentrations corresponding to the amount of particles at 10 μg/cm^2^. 2,3,7,8-Tetrachlorodibenzo-*p*-dioxin (TCDD, > 99% purity) was originally obtained from Dow Chemicals Co. (Midland, MI). Dimethylsulfoxide and phorbol-12-myristate-13-acetate (TPA) were obtained from Aldrich Chemical Co. (St. Louis, MO). Other molecular biological reagents were purchased from Qiagen (Valencia, CA) and Roche (Indianapolis, IN).

### Cell culture and differentiation.

We obtained human U937 monocytic cells from the American Tissue Culture Collection (Manassas, VA) and maintained them in RPMI 1640 medium containing 10% fetal bovine serum (Gemini, Woodland, CA), 100 U penicillin, and 100 μg/mL streptomycin supplemented with 4.5 g/L glucose, 1 mM sodium pyruvate, and 10 mM HEPES. Cell culture was maintained at a cell concentration between 2 × 10^5^ and 2 × 10^6^ cells/mL. For differentiation into macrophages, U937 cells were treated with TPA (5 μg/mL) and allowed to adhere for 48 hr in a 5% CO_2_ tissue culture incubator at 37°C, after which they were fed with TPA-free medium.

### Cell viability assay.

To assess the effect of particles on viability of U937 macrophages, we used the trypan blue exclusion test (McAteer and Davis 1994). A 10-μL portion of re-suspended cell pellet was placed in 190 μL PBS with 200 μL trypan blue (0.5% dilution in 0.85% NaCl) added. After 5 min we loaded 10 μL of the cell suspension into a hemocytometer and determined the proportion of nonviable to viable cells.

### Cellular cholesterol and protein determinations.

We extracted free and esterified cholesterol (total cholesterol) directly from macrophage monolayers *in situ* in the cell culture dish. After the indicated time of treatment, each PBS-washed monolayer was scraped off in 400 μL RI PA buffer and incubated for 30 min on ice. Unsoluble material was removed by centrifugation at 12,000 × *g* for 20 min at 4°C and aliquots were used for protein determination according to Bradford (1976). We determined the amount of free and esterified cholesterol (total cholesterol) using a colorimetric method (Roche) in the presence of cholesterol oxidase and cholesterol esterase and then measured the absorbance at 405 nm.

### Quantitative real-time reverse transcription–PCR.

We isolated total RNA from U937 cells using a high-pure RNA isolation kit (Roche) and carried out cDNA synthesis as previously described (Vogel et al. 2004b). Quantitative detection of β-actin and differentially expressed genes was performed with a LightCycler Instrument (Roche Diagnostics, Mannheim, Germany) using the QuantiTect SYBR Green PCR Kit (Qiagen) according to the manufacturer’s instructions. DNA-free total RNA (1.0 μg) was reverse-transcribed using 4 U Omniscript reverse transcriptase (RT; Qiagen) and 1 μg oligo(dT)_15_ in a final volume of 40 μL. The primers for each gene (Table 1) were designed on the basis of the respective cDNA or mRNA sequences using OLIGO primer analysis software provided by Steve Rozen and Whitehead Institute/MIT Center for Genome Research (Rosen and Skaletsky 2000), so that the targets were 100–200 bp in length. PCR amplification was carried out in a total volume of 20 μL, containing 2 μL cDNA, 10 μL 2 × QuantiTect SYBR Green PCR Master Mix, and 0.2 μM of each primer. The PCR cycling conditions were 95°C for 15 min followed by 40 cycles of 94°C for 15 sec, 60°C for 20 sec, and 72°C for 10 sec. We performed detection of the fluorescent product at the end of the 72°C extension period. We ran negative controls concomitantly to confirm that the samples were not cross-contaminated. A sample with DNase- and RNase-free water instead of RNA was concomitantly examined for each of the reaction units described above. To confirm the amplification specificity, we subjected the PCR products to melting curve analysis. We performed all PCR assays in triplicate. The intra-assay variability was < 7%. For quantification we analyzed data with the LightCycler analysis software. The variables were examined for one-sided Student’s *t* test. The results are given as the mean ± the SDs of the mean.

### Antibodies and Western blotting.

Monoclonal anti-human CRP antibody was purchased from Sigma Chemical Co. Rabbit polyclonal anti-human actin and a horseradish peroxidase-conjugated secondary antibody were obtained from Santa Cruz Biotechnology (Santa Cruz, CA). We separated whole-cell proteins on a 10% SDS–polyacrylamide gel and blotted them onto a PVDF (polyvinylidene fluoride) membrane (Immuno-Blot; BioRad, Hercules, CA). The antigen-antibody complexes were visualized using the chemoluminescence substrate SuperSignal West Pico (Pierce, Rockford, IL) as recommended by the manufacturer. For quantitative analysis, we quantified respective bands using a ChemiImager 4400 (Alpha Innotech Corp., San Leandro, CA).

### Statistical analysis.

All experiments were repeated a minimum of 3 times and results were expressed as mean ± SD. We determined statistical differences using Student’s *t* test and for the analysis of the significance between pairs of mean values, we used the Bonferroni post-hoc test.

## Results

### Dose-dependent effect of DEP and UDP on inflammatory factors and CYP1a1 expression.

To address the dose-dependent effect of the particles, we studied the mRNA expression of inflammatory factors and CYP1a1 24 hr after treatment with various concentrations of DEP and UDP. As shown in Table 2, treatment of U937 macrophages with the native particles of DEP or UDP in the range of 2.5, 10, or 40 μg/cm^2^ cell culture area led to dose-dependent mRNA induction of COX-2, TNFα, IL-6, IL-8, CRP, C/EBPβ (CCAAT/enhancer binding protein), and CYP1a1.

Except for *COX-2*, the UDP-induced expression on these genes was more prominent than the effect of DEP. All parameters were significantly induced by UDP at the low concentration of 2.5 μg/cm^2^. In contrast, COX-2, IL-6, C/EBPβ, and CRP were significantly increased only at 10 or 40 μg/cm^2^ DEP. The most conspicuous effect of UDP was found in the case of IL-8 (31.6-fold), whereas DEP showed the strongest effect on CRP expression (19.7-fold). To estimate the toxic potency, we compared the effects of DEP and UDP with various concentrations of TCDD, which has been shown to be an efficient inducer of inflammatory factors and foam-cell formation in U937 macrophages. Except for IL-6, which was downregulated by TCDD, we observed concentration-dependent increases of COX-2, TNFα, and IL-8, showing a 5.1-, 2.8-, and 2.5-fold increase, respectively, at the lowest concentration of TCDD tested (0.1 nM). CRP was also significantly increased at higher concentrations of TCDD (1 and 10 nM), which correlated with the induction of C/EBPβ.

### Evaluation of cytotoxicity of DEP and UDP.

**Figure 1:**
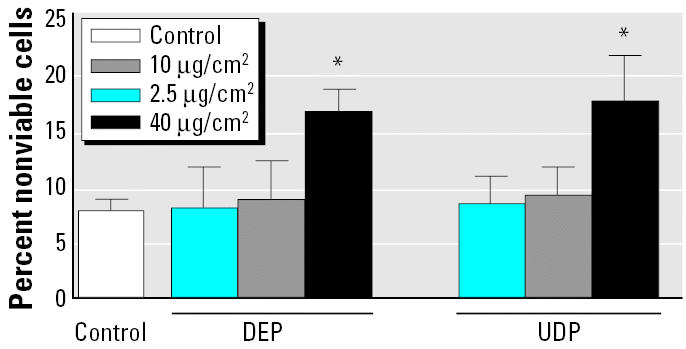
Cytotoxicity of U937 macrophages exposed to DEP or UDP. Percent cytotoxicity was assessed by trypan blue exclusion test. Cells were exposed to DEP or UDP at various concentrations (2.5, 10, or 40 μg/cm^2^). Control cells (Ctrl) received 100 μL PBS only. Error bars represent mean ± SD of three independent experiments. *Significantly different from control cells (*p* < 0.05)

Cytotoxic effects on U937 macrophages were measured for DEP, UDP, sDEP, sUDP, as well as for their corresponding extracts OE-DEP and OE-UDP. The viability of cells cultured in medium alone with 100 μL PBS added served as control. After incubating the cells with particles or extracts for 24 hr at 37°C, we determined cytotoxicity by trypan blue exclusion test. Cell death in the unexposed U937 macrophages was 8% (Figure 1). In U937 macrophages treated with 2.5 or 10 μg/cm^2^ DEP, UDP, sDEP, sUDP, OE-DEP, or OE-UDP, no significant effect on cell viability compared to control cells was found (data not shown). However, at the highest dose of 40 μg/cm^2^, treatment with DEP as well as with UDP led to a significant increase of the number of dead cells by 7 and 10%, respectively (Figure 1).

### *Effect of organic compounds in DEP- and UDP-induced expression of proinflammatory marker genes* COX-2*,* TNFα*, and* IL-8.

**Figure 2:**
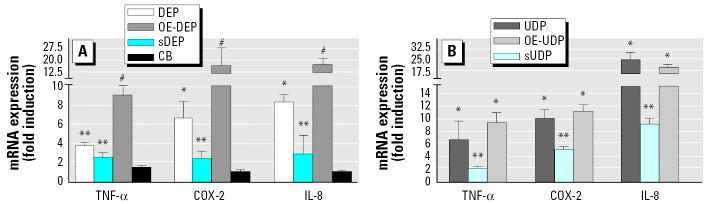
Effect of DEP (*A*), UDP (*B*), their corresponding stripped particles, or their organic extracts preparation on TNFα, COX-2, and IL-8 mRNA expression in U937 macrophages. Increased mRNA levels of TNFα, COX-2, and IL-8 are shown. (*A*) U937 macrophages were treated for 24 hr with 10 μg/cm^2^ DEP. To examine the effect of the stripped particles and the organic components of these particle samples, cells were treated with equivalent amounts of the corresponding sDEP or OE-DEP. As a control, cells were treated with 10 μg/cm^2^ CB. (*B*) U937 macrophages were treated for 24 hr with 10 μg/cm^2^ UDP. To examine the effect of the stripped particles and the organic components of these particle samples, cells were treated with equivalent amounts of the corresponding sUDP or OE-UDP. Values are given as mean ± SD of triplicates of three independent experiments. *Significantly increased compared to control cells (*p* < 0.05). **Significantly lower than in native particle-treated cells (*p* < 0.05). ^#^Significantly increased compared to native particles-treated cells (*p* < 0.05)

Macrophages involved in atherosclerotic lesions are a primary source of inflammatory cytokines. Cytokines can contribute to initiation and progression of atherosclerotic lesions by triggering multiple cellular functions such as leukocyte recruitment and synthesis/degradation of extracellular matrix. Therefore, we tested the expression of selected biomarkers of the proinflammatory response after treatment with DEP and UDP. To determine which component of DEP and UDP is the most critical one for this response, the DEP-and UDP-induced mRNA expressions were compared with those induced by sDEP, sUDP, and their organic components OE-DEP and OE-UDP. Both types of particles were used at a concentration of 10 μg/cm^2^, and the final concentration of the organic extracts was equivalent to 10 μg/cm^2^ of the particles. Treatment for 24 hr with DEP and UDP significantly induced a 7.5- and 10-fold increase of COX-2, a 4- and 7-fold increase of TNFα, and 8- and 25-fold increase of *IL-8*, respectively (Figure 2). The increases of COX-2, TNFα, and IL-8 mRNA levels by the organic compounds from the diesel particulates (OE-DEP) were more than 2-fold higher than the effect of the native particle DEP (Figure 2A), whereas the organic compounds of the urban dust particulates (OE-UDP) led to increases of these cytokines comparable to the native particle UDP (Figure 2B). For the stripped particles, sDEP, the effects on COX-2, TNFα, and IL-8 mRNA expression remained 3-fold lower than that induced by DEP, and about 8-fold lower than that induced by their organic compounds, suggesting the role of organic compounds in these responses (Figure 2A). The increase of COX-2, IL-8, and TNFαmRNA after treatment with sUDP was about 2-fold less pronounced than the effects of UDP or the organic extract OE-UDP (Figure 2B). Treatment with CB (10 μg/cm^2^) had no significant effect on the mRNA expression of COX-2, TNFα, or IL-8 (Figure 2A).

### *Role of the particles on DEP- and UDP-induced expression of* CRP *and* IL-6.

**Figure 3:**
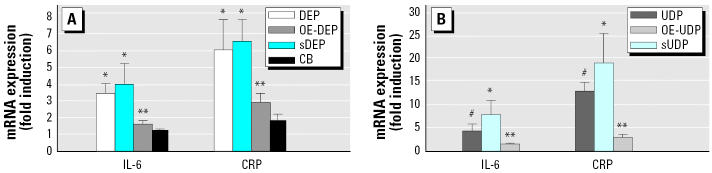
Effect of DEP (*A*), UDP (*B*), their corresponding stripped particles, or their organic extracts preparation on IL-6 and CRP mRNA expression inflammatory mediators in U937 macrophages. Increased mRNA levels of IL-6 and CRP are shown. (*A*) U937 macrophages were treated for 24 hr with 10 μg/cm^2^ DEP. To examine the effect of the stripped particles and the organic components of these particle samples, we treated cells with equivalent amounts of the corresponding sDEP or OE-DEP. As a control, cells were treated with 10 μg/cm^2^ CB. (*B*) U937 macrophages were treated for 24 hr with 10 μg/cm^2^ UDP. To examine the effect of the stripped particles and the organic components of these particle samples, cells were treated with equivalent amounts of the corresponding sUDP or OE-UDP. Values are given as mean ± SD of triplicates of three independent experiments. *Significantly increased compared to control cells (*p* < 0.05). **Significantly lower than in native particle-treated cells (*p* < 0.05). ^#^Significantly increased compared to native particle-treated cells (*p* < 0.05)

CRP participates in the systemic response to inflammation and is an important cardiovascular risk factor. Elevated concentrations of PM have been shown to be associated with increases of serum CRP levels in men (Peters et al. 2001; Pope et al. 2004b). In the present study we observed a 6- and 13-fold increase of CRP mRNA expression after exposure to 10 μg/cm^2^ DEP or 10 μg/cm^2^ UDP for 24 hr, respectively (Figure 3). Treatment with DEP or sDEP resulted in similar induction rates of CRP mRNA (6-fold), but OE-DEP had a significantly lesser effect (2.5-fold) on CRP mRNA expression than DEP or sDEP (Figure 3A). sUDP led to significantly higher induction (22-fold) of CRP mRNA than did UDP or the organic compounds OE-UDP (3-fold) (Figure 3B).

**Figure 4:**
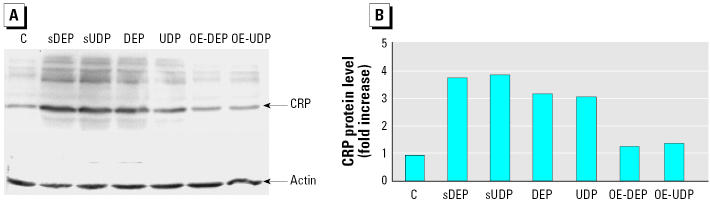
Increased intracellular protein level of CCRP. (*A*) The levels of CRP in whole-cell lysates from U937 macrophages 48 hr after treatment with 10 μg/cm^2^ DEP, UDP, sDEP, sUDP, OE-DEP, OE-UDP, or 1% PBS as vehicle control (C) were determined by Western blot analysis. Equivalent amounts of whole-cell lysates (100 μg protein) were loaded in each lane on 10% SDS-polyacrylamide gels and analyzed by immunoblotting using a CRP- or actin-(housekeeping protein as control) specific antibody. (*B*) Densitometric evaluation of CRP protein band intensities normalized to actin protein band intensities. Mean values of two independent experiments are shown.

To test whether elevated mRNA levels correspond with increased accumulation of the CRP protein, we performed Western blots. In whole-cell protein of U937 macrophages, we observed 3- to 4-fold higher protein levels of CRP in DEP-, UDP-, sDEP-, and sUDP-treated cells compared to control, OE-DEP–, or OE-UDP–treated cells (Figure 4).

IL-6 has been shown to be elevated after exposure to particles in macrophages in numerous studies (e.g., Monn and Becker 1999) and is the most potent inflammatory cytokine for the induction of human CRP. We found that after 24 hr, DEP, UDP, and their corresponding stripped particles significantly increased the mRNA level of IL-6 by 5- and 7-fold, respectively (Figure 3). However, similar to the results found for CRP, the organic compounds OE-DEP or OE-UDP did not significantly induce mRNA expression of IL-6 (Figure 3). Treatment with CB (10 μg/cm^2^) had no significant effect on the mRNA expression of IL-6. Only the CRP mRNA level was slightly elevated after exposure to CB; however, the effect was not statistically significant (Figure 3A).

The fact that treatment with TCDD, OE-DEP, or OE-UDP did not increase IL-6 and led to only a moderate increase of CRP mRNA expression compared to sDEP, sUDP, DEP, or UDP suggests that particles rather than the co-pollutants mediate the increase of CRP and IL-6.

### *Effect of various inhibitors on DEP- and UDP-mediated* CRP *and* COX-2 *induction.*

**Figure 5:**
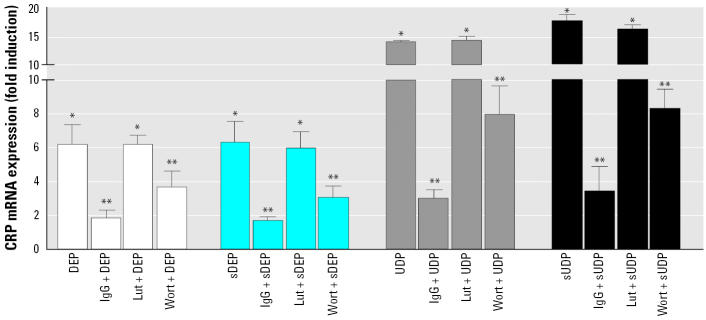
Effect of various inhibitors on DEP- or UDP-induced mRNA expression of CRP. U937 macrophages were pretreated for 15 min with 100 μg/mL aggregated human IgG, 10 μM Lut, or 0.1 μM wortmannin (Wort). Cells were then treated with 10 μg/cm^2^ DEP, UDP, or sDEP, sUDP for 24 hr. The mRNA expression was analyzed by real-time reverse transcription-PCR and results were normalized to β-actin and given as fold increase compared to the mRNA level in control cell (= 1). Values are given as mean ± SD of triplicates of three independent experiments. *Significantly increased compared to control cells (*p* < 0.05). **Significantly lower than in cells treated with native particles, stripped particles, or their organic extracts (*p* < 0.05)

**Figure 6:**
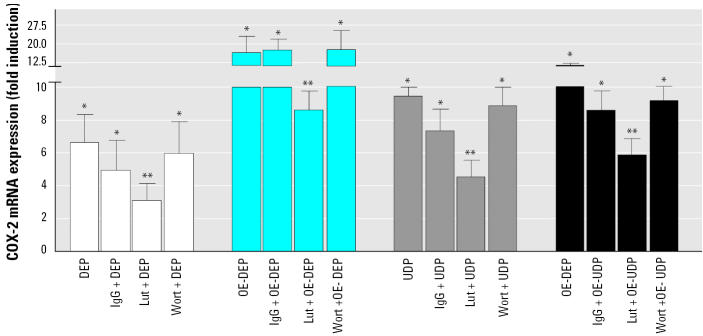
Effect of various inhibitors on DEP- or UDP-induced mRNA expression of COX-2. U937 macrophages were pretreated for 15 min with 100 μg/mL aggregated human IgG, 10 μM luteolin (Lut), or 0.1 μM wortmannin (Wort). Cells were then treated with 10 μg/cm^2^ DEP, UDP or OE-DEP, OE-UDP for 24 hr. The mRNA expression was analyzed by real-time RT-PCR and results were normalized to β-actin and given as fold increase compared to the mRNA level in control cell (= 1). Values are given as mean ± SD of triplicates of three independent experiments. *Significantly increased compared to control cells (*p* < 0.05). **Significantly lower than in cells treated with native particles, stripped particles, or their organic extracts (*p* < 0.05)

*CRP* induction by DEP, UDP, sDEP, or sUDP was blocked by about 75% after pretreatment for 15 min with 100 μg/mL aggregated IgG, which blocks binding to the Fcγ receptor. Preincubation for 15 min with 100 nM wortmannin, which inhibits Fcγ receptor–dependent ingestion and activation, blocked the induction of *CRP* mediated by DEP, UDP, sDEP, and sUDP by about 50% (Figure 5). Neither aggregated IgG nor wortmannin led to a significant inhibition of DEP- or UDP-mediated *COX-2* induction (Figure 6). Conversely, the aryl hydrocarbon hydroxylase (AhR) antagonist luteolin had no significant effect on the particle-induced expression of CRP (Figure 5). However, the induction of *COX-2* mediated by DEP/UDP, as well as their organic extracts, was significantly suppressed (50%) by luteolin (Figure 6).

### DEP- and UDP-induced CYP1a1 mRNA level.

**Figure 7:**
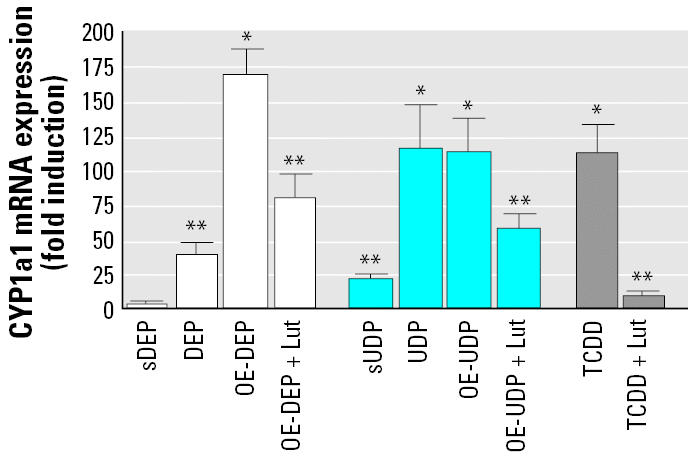
Effect of the AhR-antagonist luteolin on DEP-, UDP-, or TCDD-induced *CYP1a1* expression. U937 macrophages were treated with 10 μg/cm^2^ DEP, UDP, sDEP, sUDP, OE-DEP, OE-UDP, or 10 nM TCDD. To antagonize AhR binding and activation, cells were co-treated with 10 μM luteolin (Lut) and OE-DEP, OE-UDP, or TCDD. After 24 hr of treatment, CYP1a1 mRNA expression was analyzed by real-time RT-PCR and results were normalized to β-actin and given as fold increase compared to the mRNA level in control cell (= 1). Values are given as mean ± SD of triplicates of three independent experiments. *Significantly increased compared to control cells (*p* < 0.05). **Significantly lower than in cells treated with native particles, their organic extracts, or TCDD (*p* < 0.05).

The CYP1a1 mRNA level increased 40-fold in DEP-treated (10 μg/cm^2^) cells, whereas the organic extract OE-DEP led to a markedly higher increase of 170-fold in comparison to control cells. Exposure to 10 μg/cm^2^ UDP- or OE-UDP led to about 100-fold elevated levels of CYP1a1 mRNA after 24 hr of treatment. The stripped particles sUDP had still significant effects and increased CYP1a1 mRNA levels by 20-fold (Figure 7).

To test the role of the AhR in OE-DEP–and OE-UDP–mediated increase of *CYP1a1*, we co-treated cells with the AhR antagonist luteolin (10 μM) and OE-DEP or OE-UDP for 24 hr. Co-treatment with luteolin (10 μM) inhibited OE-DEP– and OE-UDP–mediated CYP1a1 induction by about 50%, which indicates the involvement of the AhR in this process. As a positive control, cells were treated with 10 nM TCDD for 24 hr and co-treated with luteolin. TCDD led to a 110-fold increase of CYP1a1 mRNA level, which was inhibited by 95% after co-treatment with luteolin (Figure 7).

### Stimulation of cholesterol accumulation in U937 macrophages by UDP and DEP.

**Figure 8:**
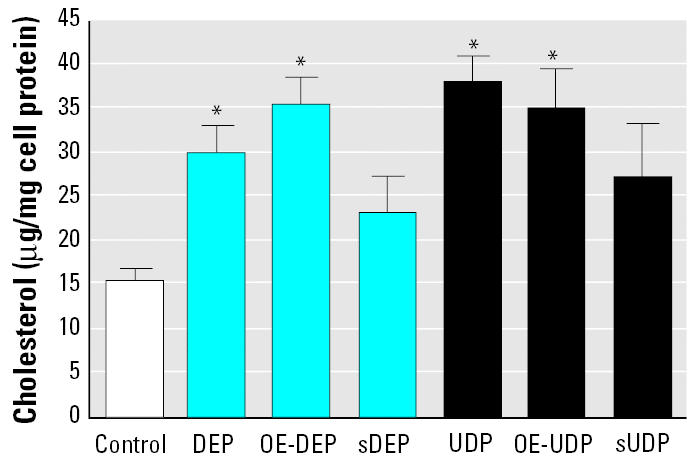
Cholesterol accumulated in U937 macrophages. Cells were treated for 5 days with 10 μg/cm^2^ DEP, UDP, sDEP, sUDP, or corresponding amounts OE-DEP, OE-UDP. Vehicle control cells received 1% PBS. Total cholesterol was determined using a colorimetric method in the presence of cholesterol oxidase and cholesterol esterase. Values are given as mean ± SD of triplicates of three independent experiments. *Significantly different from vehicle control (*p* < 0.05)

Foam cells are primarily macrophages laden with cholesterol ester-rich cytoplasmic lipid inclusions. To quantify total amount of cholesterol in U937 macrophages, we used a colorimetric method in the presence of cholesterol oxidase and cholesterol esterase. Exposure for 5 days to 10 μg/cm^2^ DEP as well as to UDP stimulated the accumulation of cholesterol by 2-and 2.5-fold, respectively (Figure 8). Both organic extracts OE-DEP or OE-UDP increased the amount of cholesterol by about 2.3-fold above control. The stripped particles sDEP and sUDP did not significantly increase the amount of cholesterol in U937 macrophages compared to control cells (Figure 8). Results from cholesterol assay were consistent with findings from Oil Red O staining (data not shown).

## Discussion

There is strong evidence from epidemiologic and animal studies that exposure to air pollution particulates play a role in the development of cardiovascular diseases such as atherosclerosis and heart diseases (Pope et al. 2004; Suwa et al. 2002). DEP and UDP, which are the most important components of PM_2.5_ (PM with aerodynamic diameter ≤2.5 μm) and PM_10_, respectively, in many urban areas, have been suspected. The results presented in this study show that diesel particles as well as urban dust cause the induction of several proinflammatory factors such as COX-2, TNFα, C/EBPβ, IL-6, and IL-8 in human macrophages. Exposure to these particles also results in a significant elevation of CRP mRNA and protein levels in U937 macrophages. We used U937-derived macrophages because these cells are frequently used to develop foam cells after treatment with modified low density protein (Martens et al. 1998) and as described earlier by exposure to environmental toxicants like TCDD (Vogel et al. 2004a). Concomitant with induction of inflammatory factors, the accumulation of total cholesterol was significantly increased in DEP-or UDP-treated macrophages. Cholesterol accumulation in macrophages is a hallmark of foam-cell formation indicating early lesion of atherosclerosis (Linton and Fazio 2003). Thus, the particle-mediated inflammatory response and subsequent formation of foam cells may contribute directly to the progression of atherosclerosis and other cardiovascular events.

Several studies have shown that the toxicity of PM might be linked to the generation of reactive oxygen species (ROS) in the lungs (Tao et al. 2003), which can be detected by their electron spin resonance signals (Kadiiska et al. 1997). ROS might also play a role in promoting a state of systemic inflammation. In the current study we performed experiments to estimate the signaling mechanisms of the proinflammatory response induced by DEP or UDP using human monocyte-derived macrophages. Organic compounds like polyaromatic hydrocarbons (PAH) adsorbed on UDP, and especially DEP, which induce *CYP1a1* gene expression (Figure 7) seemed to be mainly involved in the response of inflammatory factors such as COX-2, IL-8, and TNFα. Besides COX-2 and TNFα, we observed, for the first time, a dose-dependent increase of IL-8 mRNA level in cells treated with the AhR ligand TCDD. Results of this study show that the organic extract of OE-DEP is significantly more effective at inducing *COX-2, IL-8,* and *TNF*α than its native particle DEP, whereas OE-UDP led to a similar increase compared to its native particle UDP. These results suggest that the organic co-pollutants are highly adsorbed by DEP and thus less bioavailable compared to UDP. The stripped particles of both diesel and urban dust had significantly less effect on the induction of *COX-2, IL-8,* and *TNF*α.

To analyze the contribution of AhR-activating compounds, we co-treated cells with the AhR antagonist luteolin and the particles or their organic extracts. Our results (Figures 5–7) clearly show that luteolin is more effective in suppressing *COX-2* or *CYP1a1* than *CRP* in all samples. In turn, both IgG and wortmannin were much better inhibitors of *CRP* than *COX-2*. These results indicate that the solvent extraction procedure could effectively separate the AhR agonists (i.e., luteolin inhibited components) from the particles. Furthermore, the stripped particles showed properties different from the solvent extracts in that affected cells showed high mRNA expression of *CRP* by treatment with stripped or native particles but not with organic extracts (Figure 3).

We have previously shown that dioxin-type chemicals are powerful inducers of inflammation in U937 macrophages (Vogel et al. 2004a), and therefore it is logical to explain the action of solvent extracts to activate *COX-2*. However, the finding that stripped particles from air pollutants selectively activate *CRP* is new. It is known that insoluble, fine particles have the property to affect Fcγ receptor activity on the macrophage membrane and thereby trigger the process of phagocytosis and uptake of low-density lipoprotein (Kleinman et al. 2003; Luo et al. 2005; Ohtsuka etal. 2000; Swanson and Hoppe 2004; Zwaka etal. 2001). The notion that the stripped particles are also acting through the Fcγ receptor is supported by our observation that aggregated IgG, the specific ligand for Fcγ receptor, is effective in suppressing the same cell response indicates that the phagocytosis of those particles is the key event accompanying the Fcγ receptor stimulating action of sDEP and sDEP, since phosphatidylinositol 3-kinase, which is sensitive to wortmannin, is a crucial factor mediating phagocytosis of macrophages (Song et al. 2004). Activation of Fcγ receptor by aggregated IgG might also trigger inhibitory signaling pathways that suppress the effects of particles on the expression of *CRP*. The effect of the ultrafine CB on CRP expression was rather small, which indicates that not only the chemical components of the PM but also other factors such as surface properties and shape affect toxicity.

One interesting aspect is that the timing of particle-mediated induction of *CRP* was correlated with elevated levels of IL-6 mRNA, which can mediate transcriptional activation of *CRP*. Both *CRP* and *IL-6* induction by PM were blocked by aggregated IgG or wortmannin, which inhibits Fcγ receptor–dependent ingestion and activation. Neither aggregated IgG nor wortmannin led to a significant inhibition of DEP-or UDP-induced *COX-2*, *TNF*α, or *IL-8*. The close relationship between IL-6 and CRP has been pointed out by many scientists (e.g., Monton and Torres 1998), but in most cases CRP production is carried out in liver as a result of stimulation by circulating IL-6. The fact that IL-6 acts as an autocrine factor to stimulate CRP production in macrophages is not well known; however, the increased synthesis and secretion of CRP, IL-6, and soluble IL-6 receptor by macrophage-derived foam cells in the arterial intima has been demonstrated (Ballou and Lozanski 1992; Jones et al. 1999; Libby 2002; Lusis 2000; Ross 1999). Recent studies also show that binding of C/EBPβ is critical for induction of *CRP* expression (Agrawal et al. 2003), which could explain the increase of CRP by TCDD, as TCDD induces the expression of *C/EBPb* (Vogel et al. 2004a; Vogel and Matsumura 2003) but not *IL-6* (Table 2). According to Du Clos and Mold (2004), CRP acts through Fcγ receptor to play important roles in infection, inflammation, and autoimmune diseases. This phenomenon of action of stripped particles deserves close attention in the future.

In conclusion, we have shown that air pollution particles have two major classes of toxic components. One is the dioxin-type AhR agonist, which is extractable by solvents, and the other type is the stripped particle, which elicits a different pattern of mRNA activation from that induced by dioxin-type chemicals. These findings may contribute to a better understanding of the differential toxicity of various constituents and sources of PM, including their chemical/biological components alone or in combination with PM. Further research is necessary to fully elucidate this mechanism of differential toxicity. Other relevant sources of PM should be collected and tested, such as those from the combustion of alternative fuels, and evaluated for their potential contribution as risk factors for cardiovascular disease in both urban and rural environments.

## Figures and Tables

**Table 1:** Primer for quantitative real-time PCR analyses.

Gene	Forward primer (5′–3′)	Reverse primer (5′–3′)
β*-actin*	GGACTTCGAGCAAGAGATGG	AGCACTGTGTTGGCGTACAG
*C/EBP*β	GACAAGCACAGCGACGAGTA	AGCTGCTCCACCTTCTTCTG
*COX-2*	TGAAACCCACTCCAAACACA	GAGAAGGCTTCCCAGCTTTT
*CRP*	ATACACTGTGGGGGCAGAAG	CCGCCAAGATAGATGGTGTT
*CYP1a1*	TAGACACTGATCTGGCTGCAG	GGGAAGGCTCCATCAGCATC
*IL-6*	GAACTCCTTCTCCACAAGCG	TTTTCTGCCAGTGCCTCTTT
*IL-8*	CTGCGCCAACACAGAAATTA	ATTGCATCTGGCAACCCTAC
*TNF*α	CAGAGGGAAGAGTTCCCCAG	CCTTGGTCTGGTAGGAGACG

**Table 2:** Dose-dependent effect of DEP and UDP on COX-2, TNFα, IL-6, IL-8, C/EBPβ, CRP, and CYP1a1 mRNA expression compared to the dose-dependent effect of TCDD.

	DEP (μg/cm^2^)			UDP (μg/cm^2^)			TCDD (nM)		
Gene	0.1	1.0	10.0	2.5	10	40	2.5	10	40
*COX-2*	1.6 ± 0.5 (ns)	5.9 ± 1.1	19.5 ± 2.4	3.4 ± 0.4	6.8 ± 1.2	12.2 ± 2.1	5.1 ± 1.0	22.8 ± 2.1	43.4 ± 5.1
*TNF*α	1.8 ± 0.2	3.8 ± 0.6	8.5 ± 1.2	2.2 ± 0.2	6.9 ± 1.6	16.1 ± 1.1	2.8 ± 0.3	6.8 ± 1.1	7.8 ± 1.5
*IL-6*	1.3 ± 0.4 (ns)	3.5 ± 0.9	4.2 ± 0.9	2.0 ± 0.3	4.8 ± 1.2	6.5 ± 0.8	1.1 ± 0.4 (ns)	0.7 ± 0.5 (ns)	0.5 ± 0.2*
*IL-8*	2.3 ± 0.5	7.5 ± 1.2	11.6 ± 0.7	2.8 ± 0.4	13.5 ± 2.1	31.6 ± 3.1	2.5 ± 0.2	14.5 ± 1.4	19.7 ± 2.2
*C/EBP*β	1.5 ± 0.4 (ns)	2.2 ± 0.6	3.7 ± 0.8	1.8 ± 0.2	2.5 ± 0.3	4.8 ± 1.0	1.6 ± 1.1 (ns)	2.1 ± 0.3	3.4 ± 0.6
*CRP*	1.4 ± 1.0 (ns)	6.1 ± 1.1	19.7 ± 3.1	2.1 ± 0.4	13.4 ± 1.1	21.8 ± 2.1	1.2 ± 0.7 (ns)	2.3 ± 0.4	3.8 ± 0.5
*CYP1a1*	8.6 ± 0.8	37.9 ± 2.1	68.5 ± 4.0	28.5 ± 3.1	110.5 ± 11.2	137.0 ± 9.8	18.8 ± 2.1	120.5 ± 11.0	250.8 ± 22.5

ns, not significant. U937 macrophages were treated with 2.5, 10, or 40 μg/cm^2^ DEP or UDP. As a positive control, cells were treated with 0.1, 1.0, or 10 nM TCDD for 24 hr and mRNA was analyzed by real-time RT-PCR. Control cells received 1% PBS or 0.1% dimethylsulfoxide. Results are normalized to β-actin and given as fold increase of the mRNA levels in treated cells versus controls (= 1). Values are given as mean ± SD of triplicates of three independent experiments. All values significantly increased compared to control cells (*p* < 0.05) unless otherwise noted.

* Significantly lower than in control cells (*p* < 0.05)
